# Effectiveness of a School-Based Intervention on the Students’ Mental Health After Exposure to War-Related Trauma

**DOI:** 10.3389/fpsyt.2019.01031

**Published:** 2020-03-26

**Authors:** Basel El-Khodary, Muthanna Samara

**Affiliations:** Kingston University, Kingston upon Thames, United Kingdom

**Keywords:** war-traumatic events, posttraumatic stress disorder, counselling program, children, adolescents, Palestine

## Abstract

**Background:**

After the war, which was conducted against Palestinian civilians in the Gaza Strip, the prevalence of posttraumatic stress disorder (PTSD) among children and adolescents has increased. The counselling department at the Ministry of Education in the Gaza Strip applied a counselling program in schools in order to alleviate the effect of exposure to war. The aim of the study is to investigate the effectiveness of the counselling program after exposure to war-traumatic events among Palestinian children and adolescents in the Gaza Strip.

**Methods:**

The sample consists of 572 students aged 12–18 years old. Of them, 331 (57.9%) were female and 241 (42.1%) were male. Traumatic events were measured by *War-Traumatic Events Checklist (W-TECh)*. PTSD was measured by the *Post-Traumatic Stress Disorders Symptoms Scale (PTSDSS)*. Anxiety symptoms were adapted from *The Anxiety Symptoms Scale*, and depression symptoms were measured by *Child Depression Inventory*. Repeated measures design was employed as the counselling program was applied in all the schools at the same time. Specifically, the data were collected from the participants before the application of the counselling program and 2 months later.

**Results:**

After applying the school-based counselling program, the prevalence of PTSD (according to DSM-V) decreased from 57.5% to 45.6% among the children and adolescents who were exposed to war-traumatic events. In addition, PTSD symptoms, and emotional, somatic and cognitive functional impairment symptoms has decreased after the implementation of the counselling program especially amongst girls.

**Conclusions:**

The school-based counselling program was effective in decreasing the PTSD symptoms among children and adolescents after the exposure to war-traumatic events.

## Introduction

More than 1 billion children under the age of 18 live in armed conflict areas (1). These conflicts occur mainly in lower- and middle-income countries, where 90% of the world's population of children and adolescents live (2). In this regard, children may have a sense of insecurity and altered daily functioning after they have been exposed to war-traumatic events (3). Children and adolescents growing up with political violence and terrorism are vulnerable to intense psychological effects (4–6), which lead to psychiatric symptomatology (7, 8). The psychiatric symptomatology varied according to the context of war that the children live in, cultural specific psychiatric symptoms, the type of the trauma, the number of ongoing traumatic and stressful events that the child has experienced, and the impact of the traumatic event on the children's mental health (9–12).

Most current research is focused on school-based programs in order to reduce symptoms and enhance resilience among children and adolescents exposed to traumatic events, whether they have clinical symptoms or not (13). The school-based programs have several advantages: They are highly accessible, an effective form for alleviating psychological distress, non-stigmatizing, and associated with positive change on the students' lives (14, 15). Moreover, attending counselling programs in schools has a positive impact on the studying and learning of the students in secondary schools (16). In addition, those who are attending school-based counselling have revealed improvements in attendance and behavior (17).

Several studies have investigated the factors that protected the children living in the war-related context through a socioecological model. These protective factors may act at several layers such as household, school, and community (18, 19). Therefore, schools are suitable context in which intervention can be performed for children who have been exposed to traumatic events. In the socioecological model, parents, teachers, and mental health practitioners take part in the intervention. As a result, children may be helped to improve their mental health resilience during and after exposure to war-related trauma (20).

School-based psychosocial structured activities, which include play therapy, drama, and movement activities, were found to enhance the wellbeing of children who were affected by conflict in Northern Uganda (21). Furthermore, Loughry et al. (22) found that structural activities such as drama, art, and puppetry had a positive impact on Palestinian children's emotional and behavioral wellbeing at the time of political conflict.

The most recent systematic review study by Jordans and colleagues (2) was aimed at assessing the developments in interventions for conflict-affected children from 2009 to 2015. The majority of interventions are group interventions, either school-based or community-based. Furthermore, 90% of the intervention programs are implemented by paraprofessionals (e.g., teachers, social workers), with 95% receiving training for the intervention. However, only 43% of the various intervention programs studied showed positive overall promotion, prevention, and/or curative effects in terms of reducing symptoms and enhancing wellbeing (2). In another systematic review of psychosocial interventions, the researchers state that there has been a paucity of rigorous studies, because the publications mainly focus on posttraumatic stress disorder (PTSD) as the outcome of the intervention and the outcome evaluation (23).

### Context of the Study

In the last war against the Gaza Strip in August 2014, Israeli forces utilized air forces, rockets, and explosive weapons in the attack. Around 2,216 Palestinians were killed, of whom 1,543 were civilians, and 10,895 were injured. In addition, 8,377 houses were completely demolished, and 23,597 houses were partially destroyed. Furthermore, more than 520,000 people were displaced (24). As a result, the consequences of exposure to war-trauma were severe. Approximately 425,000 children were in need of psychosocial support; many of them needed focused counselling sessions (25). Accordingly, the counselling department of the Ministry of Education in Gaza designed a psychosocial support program.

The intervention was performed in schools and conducted following the socioecological paradigm. The intervention activities were similar for all age groups, and the number of students in each group ranged between 4 and 10 according to the type of the activity of the group. The activities have been chosen by the counsellor and the children participated in all the activities. Therefore, there is no chance for researcher bias. Those who carried out the activities including teachers, social workers, and counsellors attended a 1-week training before the start of the school. Professional counsellors from the Counselling Department at the Ministry of Education in the Gaza Strip trained them on psychosocial support program, while parents received exact and detailed instructions about the counselling program.

The short psychosocial support program was carried out over 1 week (five continuous days, 4 h per day). It consisted of specific cognitive behavioral techniques, including psychoeducation and speaking about past traumatic experiences along with the group, expressive elements, such as structural movements (e.g., physical exercises), cooperative games, and drama (see **Table 1**). The general aim of the intervention is to assist the students to stabilize themselves and feel safe as reasonably possible in the existing difficult circumstances. The intervention aims to reduce the level of specific psychological symptoms among children who are exposed to ongoing war trauma and accordingly improve the level of functioning.

**Table 1:** Psychosocial Support Program Activities.

Activities	
Day 1	- Welcoming the students and their parents and providing psychosocial support at the morning line through the school radio (psychoeducation about trauma exposure, symptoms, and consequences was provided through this activity). - Survey of traumatized and injured students (full explanation about the survey and the aim of it was provided by the counsellors). - Storytelling activity from students and parents. The aim of this activity is to let the students and their parents to express their feelings and emotions about the traumatic event.
Day 2	- Providing extracurricular activities and strengthening religious and positive attitudes and values. These can play a significant role in decreasing the effect of exposure to traumatic events. - Provision of free drawing activity for students followed by discussion of their feelings about their drawings. - Establishment of students' committees for volunteering works in each class.
Day 3	- Providing extracurricular activities and strengthening positive attitudes and values. - Providing physical activities. - Applying psychodrama and role play activities (parents actively participated in these activities). - Volunteer work activities with students, teachers, parents, and social workers (e.g., visiting injured people in hospitals, families of those who have lost loved ones in the war).
Day 4	- Open meetings for parents, teachers, counsellors, headteachers, and social workers with local organizations working on social issues. Survey of severely traumatized cases. - Variety of activities (quizzes, poems, art activities).
Day 5	- Conclusion of the activities: open-day exhibit displaying students' art works.

The aim of the current study is to investigate the effectiveness of the psychosocial support counselling program with children who were exposed to war trauma. To the best of our knowledge, this is the first study investigating the effectiveness of this program, which was applied immediately after the 2014 war on children who experienced continuous exposure to war trauma. The research questions of this study are as follows:

Has the psychosocial support counselling program had a positive effect in reducing the symptoms of exposure to war trauma?

Are there any differences in the effectiveness of the psychosocial support counselling program related to age or gender?

## Method

### Participants

The sample consisted of 572 students aged 12–18 years old (M = 14.37, SD = 1.28). Of these, 331 (57.9%) were female and 241 (42.1%) were male. The entire sample attended a psychosocial support counselling program led by trained school counsellors, social workers, and teachers. The participants were recruited from government schools in the Gaza Strip.

### Design and Sampling

This study is a longitudinal pretest and posttest experimental design (single subject design).

The Gaza Strip, where the study was conducted, consists of five governorates (Rafah, Khan Younis, Middle Area, Gaza, and North Gaza), which are referred to as places of residence. Stratified random sampling was used to choose the participants according to place of residence, type of school (primary or secondary), and gender. Primary schools include students from year 1 to year 9, while secondary schools include those from year 10 to year 12. From each place of residence, two types of schools (one primary and one secondary) were randomly chosen. From these schools, one boy school and one girl school were randomly chosen, with one class from each school being selected. Hence, 30 classes (10 classes from year 7, 10 classes from year 8, and 10 classes from year 10) were chosen on the basis of five boy classes and 5 girl classes from each year. Hence, the total number of classes was 30 (5 [place of residence] * 3 [year 7, year 8, and year 10] * 2 [male and female]).

### Ethical Procedure

Children and adolescents were given information sheets about the study and a parental consent form to give to their parents. Ethical approval was gained from the Ministry of Education in the Gaza Strip and the ethical committee at Kingston University London, UK.

### Data Collection

In order to collect the data, 30 social workers and school counsellors were trained by the researcher to carry out the study instruments with the children and adolescents in the chosen schools. After obtaining consent forms from parents, teachers, headmasters, and the Ministry of Education, the social workers and school counsellors went to the schools and administered the questionnaires to the selected students, after getting consent from the students themselves. The social workers and school counsellors explained the nature of the study, its purpose, and how the students could respond to the questionnaires. Self-reported questionnaires and interviews were utilized to collect data from the participants. The participants completed the questionnaires in two separate sessions, with each lasting approximately 40 min. The data were collected in September 2014, 1 month after the 51-day war that was conducted against the people in the Gaza Strip (pre-intervention) and 2 months after the intervention (3 months after the war).

### Study Instruments

Interviews with children and adolescents were conducted in schools to collect the data.

*Demographic variables* included age (12–18 years old), which was categorized into three groups [(1) youngest age group, less than 13 years old; (2) middle age group, 13–14 years old; and (3) oldest age group, 15 years old or more]; gender (male, female); family order (the first, the middle, the last); family size (below or above six members, being the average household size in the Gaza Strip) (26); type of residence (city, camp, village); parents' education (no school education, school education, higher education); parents' job (employed, unemployed); citizenship (refugee, not refugee); whether parents are alive or dead; and family income (below or above US $600, as the poverty line in Palestine for a household with five members is US $600) (26).

*War-Traumatic Events Checklist (W-TECh)*: (27–29): Some of the items were adapted from Gaza Traumatic Events Checklist (30), the Trauma Questionnaire Scale (31), the Gaza Traumatic Events Checklist (32), and the Checklists of Traumatic Experiences (33). The measure was modified to include items that reflected the last traumatic war events that happened in the Gaza Strip. The W-TECh consists of 30 items that cover war-traumatic experiences, including human traumatic losses or injuries and home demolition to adapt it to the nature of the last war. The items about home demolition that occurred in the last war in 2014 were as follows: Have you seen your neighbors' house destroyed by shelling or bulldozing? Have you seen other buildings destroyed by shelling or bulldozing? The W-TECh is divided into three categories: (1) experiencing personal trauma, in which children or adolescents are the target of war-related traumas, such as being shot or injured with live ammunition; (2) witnessing human trauma, in which children or adolescents witnessed others (e.g., family member, friend, or neighbor) being shot and/or injured during the war; and (3) seeing the demolition of property, in which children or adolescents observe the demolition of their home, school, and/or farm during the war. A pilot study was conducted to ensure the validity and reliability of the W-TECh. The results reveal that there is a significant correlation between the exposure to personal trauma items and the total domain (r = .269 to.524, P < .01), the witnessing trauma to others items and the total domain (r = .244 to.804, P < .01), and the seeing property demolition items and the total domain (r = .265 to 768, p < .01). Internal consistency reliability tests were run on the domains of W-TECh and the total scale. The personal domain consisted of 12 items (Cronbach's alpha = .646), the witnessing trauma to others domain consisted of 10 items (Cronbach's alpha = .767), the seeing properties demolition domain consisted of 8 items (Cronbach's alpha = .614), and the total scale consisted of 30 items (Cronbach's alpha = .830). As a result, the scale exhibited very good reliability and could be used in the study. Children and adolescents respond to the W-TECh by indicating whether they have experienced the traumatic event or not.

*Post-Traumatic Stress Disorders Symptoms Scale (PTSDSS)* adapted from 33): This scale consists of 50 items and was modified to include the diagnostic criteria of PTSD according to DSM-V, which include intrusion symptoms, avoidance, negative alterations in cognitions and mood, as well as alterations in arousal and reactivity. Functional impairment is also measured and includes items related to somatic symptoms (e.g., I get tired easily), cognitive symptoms (e.g., I cannot stop thinking about the traumatic event that I was exposed to), emotional symptoms (e.g., I get tense and nervous easily without good reason), social symptoms (e.g., I like to break the rules of my family or school), and academic dysfunction symptoms (e.g., I cannot concentrate on my studies). Children and adolescents rated their experiences on a five-point Likert scale (very often, often, moderately, rarely, or never).

Participants are considered to have PTSD when they a) have been exposed to at least one traumatic event as measured by the W-TECh; b) score moderately to very often on symptoms of at least one intrusion symptom, at least one avoidance symptom, at least two negative alterations in cognition and mood symptoms, and at least two symptoms related to alterations in arousal and reactivity; c) show significant alteration in functional impairment; and d) the duration of symptoms is more than 1 month (34). The reliability Cronbach's alpha of this measure is .94.

*The anxiety symptoms scale* (35): This is a brief self-report scale assessing generalized anxiety disorder (GAD-7). The participants completed the questionnaire by ticking the box that reflected their responses on a four-point Likert-type scale, as nearly every day = 3, more than half the days = 2, several days = 1, and not at all = 0. GAD-7 average severity interpretation: 0–4 none–minimal, 5–9 mild, 10–14 moderate, 15–21 severe. Cronbach's alpha for the anxiety scale was very good at .83.

*Child Depression Inventory* (36): This is based on the shorter version, which included 10 items, with an additional one on harming the self-included after the piloting, and each item consists of three sentences as responses. For example, the item “feeling of sadness” is represented by three sentences: I am sad once in a while; I am sad many times; and I am sad all the time. The participants responded by choosing one of the three sentences for all 11 items. The Cronbach's alpha is .801.

### Statistical Analysis

A *T*-test was used to investigate the differences between two variables (e.g., male and female) and one-way ANOVA was used to examine the differences among three or more variables (e.g., type of residence: city, camp, or village). Mixed-design repeated measures ANOVA was used with a within-subjects factor of time, i.e., pre- vs. post-intervention, for the between-subject factors of gender (male vs. female) and covariate of age (youngest age group vs. middle age group vs. oldest age group) in order to investigate the differences in the effects of the counselling program. In order to determine the effect size, the guideline interpretation of Hedge's *g* (37) was utilized, where a small effect size is considered as being 0.20, a medium effect size is 0.50, and a large effect size is 0.80 or more.

## Results

### Prevalence of the Exposure to War-Traumatic Events, PTSD, and Functional Impairments

The results show that almost every child or adolescent (99.1%, *N* = 567) had experienced at least one war-traumatic event (*M* = 11.32, *SD* = 5.25). The most prevalent war-traumatic events were witnessing or hearing shelling by tanks, artillery, or military planes (89.3%, *N* = 511); witnessing the signs of shelling on the ground (83.4%, *N* = 477); witnessing neighbors' houses being destroyed (69.2%, *N* = 396); witnessing the injury or killing of someone by the occupying forces (66.4%, *N* = 380); and witnessing a friend, a neighbor, or a close relative being killed by the occupying forces (62.8%, *N* = 359). The least prevalent war traumatic events were shooting with live ammunition by the occupying forces (4.2%, *N* = 24); being beaten by the occupying forces (5.2%, (*N* = 30); witnessing one's house being destroyed completely (7.9%, *N* = 45); being injured (e.g., wounds, burns, or bone breaking) by shelling, tanks, artillery, or military planes (9.1%, *N* = 53); and being injured to the degree that one lost consciousness (9.3%, *N* = 53). The war-traumatic events were divided into three categories and the results reveal that about 97.6% (*N* = 585) had experienced personal trauma, 92.2% (*N* = 525) had witnessed trauma to others, and 96.9% (*N* = 554) had seen the demolition of property during the war (see **Table 2**).

**Table 2:** War Traumatic Events Frequency.

No.	Items	N	%
1.	Has your house been destroyed completely by shelling or bulldozing?	45	7.9
2.	Has your house been destroyed partially by shelling or bulldozing?	149	26
3.	Have you seen your neighbors' house destroyed by shelling or bulldozing?	396	69.2
4.	Have you seen other buildings destroyed by shelling or bulldozing?	298	52.1
5.	Have you been shot with live ammunition by occupied forces?	24	4.2
6.	Have you been injured (e.g., wounds, burns, or bone break) by shelling, tanks, artillery, or military planes?	52	9.1
7.	Have you been injured to the degree that you lost consciousness?	53	9.3
8.	Have you been exposed to live fire by occupied forces, but you were not injured?	73	12.8
9.	Have you been beaten by occupied forces?	30	5.2
10.	Have you been exposed to shelling by tanks, artillery, or military planes, but you were not injured?	291	50.9
11.	Have the occupied forces sieged your house, block, camp, or zone?	81	14.2
12.	Has someone of your family members been killed by occupied forces?	72	12.6
13.	Has someone of your friends, neighbors, or close relatives been killed by occupied forces?	359	62.8
14.	Has someone of your family members been injured by occupied forces?	113	19.8
15.	Has someone of your friends, neighbors, or close relatives been injured by occupied forces?	305	53.3
16.	Have you attended to martyr's funeral?	215	37.6
17.	Have you been forced to leave your home during the war?	319	55.8
18.	Have you been detained at home during incursion?	244	42.7
19.	Have you been threatened to death by being used as human shield to arrest your neighbors by the army?	60	10.5
20.	Have you witnessed the occupied forces destroying house(s)?	322	56.3
21.	Have you witnessed or heard shelling by tanks, artillery, or military planes?	511	89.3
22.	Have you witnessed or heard the occupied forces opening fire against people?	199	34.8
23.	Have you witnessed the occupied forces beating someone?	183	32
24.	Have you witnessed killing or injuring someone by the occupied forces?	380	66.4
25.	Have you witnessed arresting someone by the occupied forces?	145	25.3
26.	Have you witnessed the occupied forces destroying trees and farms?	222	38.8
27.	Have you witnessed the occupied forces not allow ambulance access to hospital?	273	47.7
28.	Have you witnessed the signs of shelling on the ground?	477	83.4
29.	Have you watched mutilated bodies?	343	60
30.	Have you witnessed assassination of people by rockets?	246	43

Of the entire sample, 57.5% (N = 329) met the PTSD criteria of DSM-V 1 month after the war in 2014; 89.2% (N = 510) had re-experience symptoms; 63.1% (N = 361) had avoidance symptoms; 90% (N = 515) had negative alterations in cognition and mood; and 78.1% (N = 447) had alterations in arousal and reactivity. Moreover, about 45.8% (N = 262) reported moderate to severe somatic symptoms; 75.5% (N = 432) reported moderate to severe cognitive symptoms; 72.1% (N = 412) reported moderate to severe emotional symptoms; 56.4% (N = 323) reported moderate to severe social symptoms; and 52.1% (N = 298) reported symptoms of moderate to severe academic dysfunction.

### Demographic Variables With Exposure to War-Traumatic Events

The relationship of various factors with exposure to traumatic war events was investigated. One-way ANOVA tests indicate that the effect of age [*F*(2,424) = 5.81, *p = *.003, $\eta_{p}^{2}$ = .027], gender [*t*(570) = 8.915, *p* < .001, *d = *.751], father's education level [*F*(2,561) = 6.39, *p = *.002, $\eta_{p}^{2}$ = .022], mother's education level [*F*(2,562) = 9.16, *p* < .001, $\eta_{p}^{2}$ = .032], and father's employment [*t*(566) = 1.97, *p = *.04, *d = *.165] were significant. Bonferroni *post hoc* tests show that the oldest age group had more exposure to war-traumatic events than the middle age group (*p = *.005). Also, boys exhibited greater exposure to war-traumatic events than girls (*p* < .001). In addition, children and adolescents whose fathers (*p = *.001) and mothers (*p* < .001) had no school education were exposed to more war-traumatic events than those whose parents had school education. Moreover, children and adolescents whose mothers had no school education were exposed to more war-traumatic events than those whose mothers had higher education (*p = *.01). Finally, children and adolescents whose fathers were unemployed reported more exposure to war-traumatic events compared to those whose fathers were employed (*p* < .04) (see **Table 3**).

**Table 3:** Demographic Variables Frequencies.

	N	%	Total trauma M (SD)
**Age** 12 or less 13–14 15 or more	11 344 197	1.9 60.1 34.4	*p* = .003 9.36 (3.95) 11.01 (5.33) 13.18 (5.13)
**Gender** Male Female	241 331	42.1 57.9	*p* < .001 13.48 (5.11) 8.20 (4.87)
**Family size** Below average Above average	61 460	10.7 80.4	*p* = .23 10.47 (5.14) 11.31 (5.20)
**Type of residence** City Refugee camp Village	397 103 59	69.4 18 10.3	*p* = .16 11.34 (5.29) 11.40 (4.86) 10.00 (5.07)
**Family income** Below average Above average	413 111	72.2 19.4	*p* = .17 11.39 (5.08) 10.64 (5.40)
**Father education** None School education Higher education	110 305 149	19.2 53.3 26	*p* = .002 12.82 (5.23) 10.75 (4.96) 11.35 (5.69)
**Father job** Unemployed Employed	274 294	47.9 51.4	*p* = .04 11.74 (5.45) 10.87 (5.03)
**Father** Alive Dead	543 22	94.9 3.8	*p* = .31 11.26 (5.22) 12.40 (6.20)
**Mother education** None School education Higher education	98 366 101	17.1 64 17.7	*p* < .001 13.30 (4.96) 10.78 (5.15) 11.26 (5.48)
**Mother job** Unemployed Employed	531 36	92.8 6.3	*p* = .15 11.22 (5.16) 12.50 (6.42)
**Mother** Alive Dead	550 22	96.2 2.4	*p* = .68 11.27 (5.23) 11.85 (4.46)
**Citizenship** Refugee Not refugee	141 416	24.7 72.7	*p = *.48 11.03 (4.66) 11.38 (5.38)

### Mental Health Diagnosis Over Time by Gender, Age and Time (Pre–Post-Intervention)

#### PTSD

Mixed ANOVA analyses show a significant effect of time on PTSD diagnosis, according to DSM-V [*χ*^2^(1, *N* = 513) = 77.63, *p* < .001]. The prevalence of children and adolescents who met the diagnostic criteria of PTSD reduced from 57.5% pre-intervention to 45.6% post-intervention. Mixed ANOVA analyses reveal a significant effect of time on PTSD symptoms [*F*(1, 511) = 15.04, *p* < .001, $\eta_{p}^{2}$ =.03]; re-experience cluster symptoms [*F*(1, 511) = 12.65, *p* < .001, $\eta_{p}^{2}$ = .024]; negative alterations in cognition and mood cluster symptoms [*F*(1, 511) = 4.35, *p = *.03, $\eta_{p}^{2}$ = .008]; somatic symptoms [*F*(1, 511) = 13.72, *p* < .001, $\eta_{p}^{2}$ = .026]; cognitive symptoms [*F*(1, 511) = 42.53, *p* < .001, $\eta_{p}^{2}$ = .077]; emotional symptoms [*F*(1, 506) = 30.00, *p* < .001, $\eta_{p}^{2}$ = .056]; and academic dysfunction symptoms [*F*(1, 505) = 5.37, *p = *.02, $\eta_{p}^{2}$ = .011]. Children and adolescents reported fewer PTSD symptoms, re-experience cluster symptoms, negative alterations in cognition and mood cluster symptoms, somatic symptoms, as well as cognitive, emotional, and academic dysfunction symptoms after the intervention compared to before. In contrast, mixed ANOVA analyses show no significance regarding time for avoidance cluster symptoms (*p = *.13), alterations in arousal and reactivity cluster symptoms (*p = *.99), and social symptoms (*p = *.21) (see **Table 4**).

**Table 4:** Means and Standard Deviations of PTSD, PTSD Clusters, Anxiety, Depression, and Functional Impairment Regarding Gender Pre- and Post-intervention.

	N (M, F)	Pre-intervention			Post-intervention		
		Male M (SD)	Female M (SD)	Total M (SD)	Male M (SD)	Female M (SD)	Total M (SD)
**PTSD (total)**	513(197, 316)	46.52(25.07)	54.99(25.24)	51.74(25.49)	44.65(24.33)	49.35(24.73)	47.55(24.66)
Re-experience	513(197, 316)	2.83(1.98)	4.06(1.77)	3.59(1.95)	2.75(2.15)	3.54(1.87)	3.24(2.02)
Avoidance	508(194, 314)	0.86(0.84)	1.06(0.85)	0.98(0.85)	0.83(.84)	0.96(0.88)	0.91(0.87)
Negative alterations in cogitations and mood	513(197, 316)	6.18(3.52)	6.66(3.57)	6.47(3.55)	6.24(3.78)	5.97(3.64)	6.07(3.69)
Alterations in arousal and reactivity	513(197, 316)	3.77(2.70)	3.93(2.51)	3.87(2.58)	4.02(2.97)	3.68(2.70)	3.81(2.80)
Somatic symptoms	513(197, 316)	11.01(7.45)	13.61(7.82)	12.61(7.78)	11.07(7.77)	11.21(6.98)	11.15(7.29)
Cognitive symptoms	513(197, 316)	19.10(9.78)	23.13(10.37)	21.58(10.32)	17.37(9.16)	19.60(9.64)	18.74(9.51)
Emotional symptoms	508(193, 315)	16.01(8.16)	22.18(8.75)	19.83(9.04)	14.91(8.27)	19.22(8.87)	17.58(8.89)
Social symptoms	509(194, 315)	16.56(11.53)	17.40(11.71)	17.08(11.64)	16.93(11.83)	15.83(11.23)	16.25(11.46)
Academic dysfunction symptoms	507(192, 315)	12.35(8.55)	10.49(8.57)	11.20(8.60)	13.28(8.64)	11.28(8.70)	12.04(8.72)
**Anxiety**	501(186, 315)	5.98(4.14)	6.73(4.70)	6.45(4.51)	6.10(4.28)	6.30(4.38)	6.23(4.34)
**Depression**	495(183, 312)	5.80(3.71)	5.67(3.51)	5.72(3.59)	6.49(3.98)	5.81(3.99)	6.06(3.99)

Mixed ANOVA analyses show a significant effect of *gender* on PTSD [*F*(1, 511) = 10.40, *p = *.001, $\eta_{p}^{2}$ = .020]; re-experience cluster symptoms [*F*(1, 511) = 43.31, *p* < .001, $\eta_{p}^{2}$ = .078]; avoidance cluster symptoms [*F*(1, 506) = 6.55, *p = *.01, $\eta_{p}^{2}$ = .013]; somatic symptoms [*F*(1, 511) = 5.13, *p = *.02, $\eta_{p}^{2}$ = 010]; cognitive symptoms [*F*(1, 511) = 15.51, *p* < .001, $\eta_{p}^{2}$ = 029]; emotional symptoms [*F*(1, 506) = 57.08, *p* < .001, $\eta_{p}^{2}$ = .10]; and academic dysfunction symptoms [*F*(1, 505) = 7.61, *p = *.006, $\eta_{p}^{2}$ = .015]. Specifically, girls reported fewer PTSD symptoms, re-experience cluster symptoms, avoidance cluster symptoms, somatic symptoms, and cognitive and emotional symptoms than boys. On the other hand, boys reported more academic dysfunction symptoms after the intervention when compared to before. Moreover, the results show no effect of gender in terms of negative alterations in cognition and mood cluster symptoms (*p = *.72), alterations in arousal and reactivity cluster symptoms (*p = *.69), and social symptoms (*p = *.89) (see **Table 4**).

Mixed ANOVA analyses show a significant effect of *age* on academic dysfunction symptoms [*F*(2, 494) = 3.37, *p = *.03, $\eta_{p}^{2}$ = .013]. A Bonferroni *post hoc* test shows that the oldest age group reported less academic dysfunction symptoms than the middle age group (*p = *.07). In contrast, mixed ANOVA analyses display no significant effect of age regarding PTSD (*p = *.40), re-experience cluster symptoms (*p = *.14), avoidance cluster symptoms (*p = *.30), negative alterations in cognition and mood cluster symptoms (*p = *.18), alterations in arousal and reactivity cluster symptoms (*p = *.15), somatic symptoms (*p *.99), cognitive symptoms (*p = *.07), emotional symptoms (*p = *.20), or social symptoms (*p = *.79).

Mixed ANOVA analyses show no significant age × gender interaction for PTSD (*p = *.89); re-experience cluster symptoms (*p = *.69); avoidance cluster symptoms (*p = *.98); negative alterations in cognition and mood cluster symptoms (*p = *.63); alterations in arousal and reactivity cluster symptoms (*p = *.93); somatic symptoms (*p = *.74); cognitive symptoms (*p = *.90); emotional symptoms (*p = *.59); social symptoms (*p = *.86); or academic dysfunction symptoms (*p = *.24).

#### Anxiety

The results show that the level of anxiety was mild before the intervention (M = 6.45) and after the intervention (M = 6.32). The results also show that there is no significant effect of time (*p = *.51); gender (*p = *.18); or gender × age interaction (*p = *.24) on the level of anxiety before and after the intervention (see **Table 4**).

#### Depression

Mixed ANOVA analyses show a significant effect of time on depression [*F*(1, 493) = 5.47, *p = *.02, $\eta_{p}^{2}$ = .011]. Children and adolescents reported more depression symptoms after the intervention compared to before the intervention. In contrast, the results show no effect of gender (*p = *.19), age (*p = *.23), or gender × age interaction (*p = *.42) on the level of depression before and after the intervention (see **Table 4**).

## Discussion

The present study was aimed at investigating the effectiveness of a short-term school-based psychosocial counselling program on the mental health of children and adolescents. The study was conducted immediately after the war that took place in July 2014 in the Gaza Strip. This is the third major war that the children experienced during the last 6 years. As a result, they have been more likely to develop mental health problems. Professionals working in the Counselling Department at the Ministry of Education in the Gaza Strip implemented a short-term school-based psychosocial counselling program for all students after the war. Parents, teachers, counsellors, and social workers have been involved and participated in the intervention. The intervention started in the first week of the academic year and was aimed at mitigating the effect of exposure to war-related traumatic events by reducing PTSD symptoms. It included specific strategies (e.g., psychoeducation, psychodrama, role play, storytelling, and free drawing) that have shown positive effects on children's wellbeing.

Previous results have shown that psychoeducation strategies (information has been given to the children about the traumatic events) assist children in restructuring themselves and having a sense of control, thereby being able to recover faster (21, 22, 38). Moreover, drama games exercises have been found to be beneficial for trauma recovery in terms of improving feelings of dysregulation and social isolation as well as enhancing the sense of power, which can be very helpful toward the recovery process after trauma exposure (38). In addition, it has been elicited that using a free drawing strategy enables children to identify the social support that they need after exposure to such an event (39). Furthermore, cooperative games, such as psychodrama, facilitate children promoting cohesion within groups and fostering cooperation (21, 22). Finally, in the intervention, the children applied the storytelling strategy, which pertains to speaking about what has disturbed them about the traumatic experience, which can let them feel better after doing so (38).

The results indicate that almost every child or adolescent had been exposed to at least one war-traumatic event. Consequently, the prevalence of exposure to each category of the war-traumatic checklist (experiencing personal trauma, witnessing trauma to others, and seeing properties demolition) was very high; at least 92.2% of the children experienced all the types of exposure. Moreover, 57.5% of the children met the diagnostic criteria of PTSD according to DSM-V. These findings are in line with previous studies, thus indicating that the greater the exposure to traumatic events, the greater the possibility of developing PTSD symptoms and diagnosis (8, 33, 40). Also, consistent with previous studies (41, 42), the results show that exposure to war-traumatic events causes functional impairment, such as cognitive, emotional, and/or social symptoms.

The results revealed that boys reported more exposure to war-traumatic events than girls. In the Palestinian culture, boys are urged to take part in the political activities (43), and accordingly, they are more liable to be exposed to traumatic events. This is in line with previous studies (44–46). Furthermore, older children reported more exposure to war-traumatic events than younger ones. Culturally, children in Palestine think that the participation in the community activities is one of their responsibilities, the sense of which increases as they get older. Accordingly, their participation in the community activities during the war increased, and hence, they became more vulnerable to being exposed to war-traumatic events.

Further, girls exhibited greater improvement in PTSD symptoms over time after the intervention, a finding in line with previous studies (47, 48). However, this differs from Qouta et al. (49), who elicited that school-based intervention had a more positive impact on boys when compared to girls. With regards to functional impairment, girls reported lower somatic symptoms, and cognitive and emotional symptoms compared to boys, while the latter reported more academic dysfunction symptoms than the former.

According to the ecological developmental theory, children's mental health is affected by their characteristics and the social context, including the family, peer, school, and the community levels. That is, these factors interact to shape the children's personality and behavior (50, 51) and play a critical role in their development (50, 52–55). Therefore, social context appears to be crucial in the implementation of the intervention for the children who develop PTSD. Consequently, positive effects from intervention can be achieved if traumatized children have a supportive environment (39). Based on the ecological theory, one of the main aims of the intervention was to include parents and teachers in its activities, which contributed positively to the children's mental health during and after the application of the activities. That is, many children reported improvement in their mental health after the application of the intervention. This also can be achieved by including organizations and centers that provide psychosocial intervention programs for students in schools and individually for those who report severe mental disorders. Future studies should also take into account employment status, educational level, time spent with children, and parental support.

Brunzell et al. (56) suggested a Trauma-Informed Positive Education (TIPE) model to deal with traumatized students. The model focuses on both defect perspective such as developmental struggles that students suffer from and strengths perspective such as psychological resources that students have to success. It is proposed that three domains are needed for traumatized children: repairing regular abilities, repairing disrupted attachment, and empowering psychological resources. In the current study, teachers were involved in the intervention program, and they have trained in some counseling activities to implement with the students. So, they can help the students who are still struggling in schools.

The results also showed that there is no significant effect of time, gender, and gender/age interaction on the level of anxiety before and after the intervention, while for depression, there was only a significant effect for time; depression increased after the intervention. These results are in line with previous studies [e.g., (57)], which indicated that classroom-based intervention has low effect in reducing anxiety and depressive symptoms. Therefore, a focused attention should be given to providing a different kind of psychosocial support for these disorders. International consensus guidelines [e.g., the Inter-Agency Standing Committee (IASC)] regarding mental health and psychological support in emergency situations agree on the necessity of a multilayered system of support delivered at various levels in the social and health systems. Moreover, they emphasize the importance of the integration of different kinds of support including: 1) activities for the population as a whole, such as providing general humanitarian support with respect to dignity; 2) nonspecialized activities that intensify protective factors for people who still need more individual or group intervention. The nonspecialized activities can be applied by trained and supervised workers (but who may not have had years of training in specialized care); and 3) intervention programs that address a smaller number of the population, who have revealed significant psychological distress or mental disorders that require more specialized support (58). These interventions should be focused more on specific disorders and symptoms such as anxiety and depression.

The study has several limitations. Firstly, the intervention was implemented for all students, so there is no control group to allow for any causal interpretation. Secondly, the post-intervention was performed only once for all students in all schools 2 months after the intervention, after which the students will go through final exams and then holidays. Thus, a longer follow-up period should be in place to measure the long-term effects of the intervention. Thirdly, the results show that anxiety did not reduce after the intervention, hence leaving a very fertile ground to the emergence of long-term discrete symptoms. Nevertheless, the study is of high importance to the understanding of relevant psychosocial activities in children and adolescents with PTSD, and more importantly, it sheds light to a warzone and a conflict context where studies are scarce. A future mixed method study (quantitative and qualitative) is needed to investigate in depth the effectiveness of this intervention. A qualitative-clinical interview study will prompt for more in-depth responses about the causes and consequences of war trauma and the benefits of particular aspects of the intervention that could help in specific PTSD criteria. Finally, the study depended on children's self-reporting, which can be a limiting factor and hence future studies should consider collecting data from other resources, such as parents and teachers. The Gaza Strip is in a severe deprivation of basic needs and is regularly exposed to continuous traumas and political instability. These aspects need to be taken into account in future studies. On the other hand, there is a need for proper diagnostic methods that suit the context of ongoing trauma and thus ongoing PTSD.

## Conclusion

School-based intervention was tailored to decrease the psychological symptoms and improve resilience among children and adolescents who had been exposed to war trauma. It was also designed to provide social support for parents and teachers in order to bring back their routine lifestyle. The intervention included strategies such as storytelling, psychodrama, and role playing, which indicated a positive effect on reducing the prevalence of a PTSD symptoms and diagnosis, according to DSM-V classification.

## Data Availability Statement

The datasets generated for this study are available on request to the corresponding authors.

## Ethics Statement

The studies involving human participants were reviewed and approved by the Ethics committee of Kingston University London, UK. Written informed consent to participate in this study was provided by the participants' legal guardian/next of kin.

## Author Contributions

BE-K conceived of the study and its design, and coordinated and drafted the manuscript. MS conceived of the study and its design, and coordinated and drafted the manuscript. All authors read and approved the final manuscript.

## Funding

This study was supported by the Qatar National Research Fund (QNRF), a member of Qatar Foundation Doha, Qatar, National Priority Research Programs (NPRP) Grant (NPRP 7-154-3-034) funded to Professor MS. We thank QNRF for their support. We also thank Qatar University and Islamic University of Gaza for providing continuous and full support and help to BE-K.

## Conflict of Interest

The authors declare that the research was conducted in the absence of any commercial or financial relationships that could be construed as a potential conflict of interest.

## References

[1] Haroz EE Jordans M de Jong J Gross A Bass J Tol W Measuring hope among children affected by armed conflict: cross-cultural construct validity of the children's hope scale. Assessment (2017) 24(4):528–39. 10.1177/1073191115612924 PMC5835958 26508802

[2] Jordans MJD Pigott H Tol WA Interventions for children affected by armed conflict: a systematic review of mental health and psychosocial support in low- and middle-income countries. Curr Psychiatry Rep (2016) 18(1):1–15. 10.1007/s11920-015-0648-z 26769198 PMC4713453

[3] Abdeen Z Qasrawi R Nabil S Shaheen M Psychological reactions to Israeli occupation: findings from the national study of school-based screening in Palestine. Int J Behav Dev (2008) 32(4):290–7. 10.1177/0165025408092220

[4] Thabet AAM Abed Y Vostanis P Comorbidity of PTSD and depression among refugee children during war conflict. J Child Psychol Psychiatry Allied Disciplines (2004) 45(3):533–542:15055372. 10.1111/j.1469-7610.2004.00243.x 15055372

[5] Khamis V Post-traumatic stress disorder among school age Palestinian children. Child Abuse Negl (2005) 29(1):81–95. 10.1016/j.chiabu.2004.06.013 15664427

[6] Khamis V Post-traumatic stress and psychiatric disorders in Palestinian adolescents following intifada-related injuries. Soc Sci Med (2008) 67(8):1199–207. 10.1016/j.socscimed.2008.06.013 18657343

[7] Dyregrov A Gupta L Gjestad R Mukanoheli E Trauma exposure and psychological reactions to genocide among rwandan children. J Traumatic Stress (2000) 13(1):3–21. 10.1023/A:1007759112499 10761171

[8] McFarlane A Biology not culture explains dissociation in posttraumatic stress disorder. [References]. Biol Psychiatry (2013) 73(4):296–7. 10.1016/j.biopsych.2012.11.026 23351887

[9] Cummings EM Merrilees CE Taylor LK Mondi CF Developmental and social–ecological perspectives on children, political violence, and armed conflict. Dev Psychopathol (2017) 29(1):1–10. 10.1017/S0954579416001061 27869066

[10] Griffith JL Kohrt B Dyer A Polatin P Morse M Jabr S Training psychiatrists for global mental health: cultural psychiatry, collaborative inquiry, and ethics of alterity. Acad Psychiatry (2016) 40(4):701–6. 10.1007/s40596-016-0541-z PMC5494286 27060095

[11] Jabr S Morse M El Sarraj W Awidi B Mental health in Palestine: country report. Arab J Psychiatry (2013) 24(2):174–8. 10.12816/0001376

[12] Kohrt BA Rasmussen A Kaiser BN Haroz EE Maharjan SM Mutamba BB Cultural concepts of distress and psychiatric disorders: literature review and research recommendations for global mental health epidemiology. Int J Epidemiol (2014) 43(2):365–406. 10.1093/ije/dyt227 24366490 PMC3997373

[13] Diab M Peltonen K Qouta SR Palosaari E Punamäki R-L Effectiveness of psychosocial intervention enhancing resilience among war-affected children and the moderating role of family factors. Child Abuse Negl (2015) 40:24–35. 10.1016/j.chiabu.2014.12.002 25534065

[14] Cooper M Counselling in UK secondary schools: a comprehensive review of audit and evaluation data. Counselling Psychother Res (2009) 9(3):137–50. 10.1080/14733140903079258

[15] Cooper M Stewart D Sparks J Bunting L School-based counseling using systematic feedback: a cohort study evaluating outcomes and predictors of change. Psychother Res (2013) 23(4):474–88. 10.1080/10503307.2012.735777 23116331

[16] Rupani P Cooper M McArthur K Pybis J Cromarty K Hill A The goals of young people in school-based counselling and their achievement of these goals. Counselling Psychother Res (2014) 14(4):306–14. 10.1080/14733145.2013.816758

[17] Pybis J Hill A Cooper M Cromarty K A comparative analysis of the attitudes of key stakeholder groups to the Welsh Government's school-based counselling strategy. Br J Guidance Counselling (2012) 40(5):485–98. 10.1080/03069885.2012.718736

[18] Veronese G Castiglioni M ‘When the doors of Hell close': dimensions of well-being and positive adjustment in a group of Palestinian children living amidst military and political violence. Childhood (2015) 22(1):6–22. 10.1177/0907568213512692

[19] Wessells MG Children and armed conflict: interventions for supporting war-affected children. Peace Conflict: J Peace Psychol (2017) 23(1):4–13. 10.1037/pac0000227

[20] Ager A Metzler J Where there is no intervention: insights into processes of resilience supporting war-affected children. Peace Conflict: J Peace Psychol (2017) 23(1):67. 10.1037/pac0000211

[21] Alegre A Parenting styles and children's emotional intelligence: what do we know? Family J (2011) 19(1):56–62. 10.1177/1066480710387486

[22] Loughry M Ager A Flouri E Khamis V Afana AH Qouta S The impact of structured activities among Palestinian children in a time of conflict. J Child Psychol Psychiatry (2006) 47(12):1211–8. 10.1111/j.1469-7610.2006.01656.x 17176376

[23] Jordans MJD Tol WA Komproe IH De Jong JVTM Systematic review of evidence and treatment approaches: psychosocial and mental health care for children in war. Child Adolesc Ment Health (2009) 14(1):2–14. 10.1111/j.1475-3588.2008.00515.x

[24] Palestinian Centre for Human Rights (2014). Annual report Ref number: 49/2015. Retrieved from: http://www.pchrgaza.org/files/2015/annual_pchr_eng_2014.pdf.

[25] UNICEF Ex-Post Evaluation of UNICEF Humanitarian Action for Children 2014-2015 in the State of Palestine. (2016) 118.

[26] Palestinian Central Bureau of Statistics Palestine in figures. Ramallah – Palestine (2014).

[27] El-Khodary B Samara M The effect of exposure to war-traumatic events, stressful life events, and other variables on mental health of Palestinian children and adolescents in the 2012 Gaza War. Lancet (2018) 391:S6. 10.1016/S0140-6736(18)30331-3 29553455

[28] El-khodary B Samara M The relationship between multiple exposures to violence and mental health and behavioural problems among Palestinian children and adolescents. J Adolescence (2019a), 1018–8827. 10.1007/s00787-019-01376-8 31352503

[29] El-khodary B Samara M The relationship between trait emotional intelligence, prosocial behaviour, parental support and parental psychological control and PTSD and depression. J Res In Pers (2019b) 81:246–56. 10.1016/j.jrp.2019.06.004

[30] Hein FA Qouta S Thabet A el Sarraj E Trauma and mental health of children in Gaza. Br Med J (1993) 306(6885):1130–1. 10.1136/bmj.306.6885.1130-c PMC1677511 8495185

[31] Qouta El-sarraj Prevalence of PTSD among Palestinian children in Gaza Strip. ArabPsynet (2004) 2, 8–13. 10.1007/s00787-003-0328-0

[32] Thabet AA Abu Tawahina A El Sarraj E Vostanis P Exposure to war trauma and PTSD among parents and children in the Gaza strip. Eur Child Adolesc Psychiatry (2008) 17(4):191–9. 10.1007/s00787-007-0653-9 18365135

[33] Altawil M Harrold D Samara M Children of war in Palestine. Children in War : The International Journal of Evacuee and War Child Studies (2008) 1(5):7–14. ISSN (print) 1745-7211.

[34] American Psychiatric Association Diagnostic and Statistical Manual of Mental Disorders. American Psychiatric Association (2013). Retrieved from http://dsm.psychiatryonline.org/doi/book/10.1176/appi.books.9780890425596.

[35] Spitzer RL Kroenke K Williams JBW Löwe B A brief measure for assessing generalized anxiety disorder: the GAD-7. Arch Internal Med (2006) 166(10):1092–7. 10.1001/archinte.166.10.1092 16717171

[36] Kovacs M Childrens Depression Inventory. Multi-Health System: New York (1992). Retrieved from: https://www.pearsonclinical.co.uk/Psychology/Generic/ChildrensDepressionInventory(CDI)/Resources/Technical.pdf.

[37] Cohen J Statistical Power Analysis for the Behavioral Sciences. L. Erlbaum Associates (1988).

[38] Macy RD Macy DJ Gross SI Brighton P Healing in familiar settings: support for children and youth in the classroom and community. New Dir Youth Dev (2003)(98), 51–79. 10.1002/yd.44 12970987

[39] Tol WA Komproe IH Jordans MJ Ndayisaba A Ntamutumba P Sipsma H School-based mental health intervention for children in war-affected Burundi: a cluster randomized trial. BMC Med (2014) 12:56. 10.1186/1741-7015-12-56 24690470 PMC3994237

[40] Khamis V Posttraumatic stress and worry as mediators and moderators between political stressors and emotional and behavioral disorders in Palestinian children. Int J Psychol (2012) 47(2):133–41. 10.1080/00207594.2011.598524 22046999

[41] Al-Krenawi A Graham JR Kanat-Maymon Y Analysis of trauma exposure, symptomatology and functioning in Jewish Israeli and Palestinian adolescents. Br J Psychiatry (2009) 195(5):427–32. 10.1192/bjp.bp.108.050393 19880933

[42] Pat-Horenczyk R Qasrawi R Lesack R Haj-Yahia M Peled O Shaheen M Posttraumatic symptoms, functional impairment, and coping among adolescents on both sides of the israeli–palestinian conflict: a cross-cultural approach. Appl Psychol (2009) 58(4):688–708. 10.1111/j.1464-0597.2008.00372.x

[43] Baker A Shalhoub-Kevorkian N Effects of political and military traumas on children: the palestinian case. Clin Psychol Rev (1999) 19(8):935–50. 10.1016/S0272-7358(99)00004-5 10547711

[44] Dubow EF Boxer P Huesmann LR Shikaki K Landau S Gvirsman SD Exposure to conflict and violence across contexts: Relations to adjustment among Palestinian children. J Clin Child Adolesc Psychology: Off J Soc Clin Child Adolesc Psychol Am psychol Assoc Division (2010) 53, 39(1):103–16. 10.1080/15374410903401153 PMC2856124 20390802

[45] Peltonen K Punamäki R-L Preventive interventions among children exposed to trauma of armed conflict: a literature review. Aggressive Behav (2010) 36(2):95–116. 10.1002/ab.20334 19998393

[46] Thabet AA Ibraheem AN Shivram R Winter EA Vostanis P Parenting support and PTSD in children of a war zone. Int J Soc Psychiatry (2009) 55(3):226–37. 10.1177/0020764008096100 19383666

[47] Bolton P Bass J Betancourt T Speelman L Onyango G Clougherty KF Interventions for depression symptoms among adolescent survivors of war and displacement in northern Uganda: a randomized controlled trial. JAMA (2007) 298(5):519–27. 10.1001/jama.298.5.519 17666672

[48] Tol WA Komproe I. H., D, J. Gross AL Susanty D Macy RD M TV Mediators and moderators of a psychosocial intervention for children affected by political violence. J Consult Clin Psychol (2010) 78(6):818–28. 10.1037/a0021348 21114342

[49] Qouta SR Palosaari E Diab M Punamäki R-L Intervention effectiveness among war-affected children: a cluster randomized controlled trial on improving mental health. J Traumatic Stress (2012) 25(3):288–98. 10.1002/jts.21707 22648703

[50] Bronfenbrenner U Toward an experimental ecology of human development. Am Psychol (1977) 32:513–31. 10.1037/0003-066X.32.7.513

[51] Bronfenbrenner U The ecology of human development. Cambridge, MA: Harvard University Press (1979).

[52] Bronfenbrenner U Ecological systems theory. In: Vasta RI, editor. Annals of child development:Vol. 6. Six theories of child development. Greenwich, CT: JAI Press (1989). p. 187–249.

[53] Bronfenbrenner U McClenlland P Wethington E Moen P Ceci S The State of Americans: This Generation and the Next. Simon and Schuster. (1996).

[54] Bronfenbrenner U Morris PA The ecology of developmental processes. In: Damon W Lerner RM, editors. Handbook of child psychology: Theoretical models of human development. (1998). p. 993–1028.

[55] Hill M Tisdall K Children & Society. Longman, Harlow (1997).

[56] Brunzell T Stokes H Waters L Trauma-informed positive education: using positive psychology to strengthen vulnerable students. Contemp School Psychol (2016) 20(1):63–83. 10.1007/s40688-015-0070-x

[57] Jordans MJD Komproe IH Tol WA Kohrt BA Luitel NP Macy RD Evaluation of a classroom-based psychosocial intervention in conflict-affected Nepal: a cluster randomized controlled trial. J Child Psychol Psychiatry Allied Discipl (2010) 51(7):818–26. 10.1111/j.1469-7610.2010.02209.x 20102428

[58] Inter-Agency Standing Committee (IASC) IASC Guidelines on Mental Health and Psychosocial Support in Emergency Settings. Geneva (2007). 10.1080/09540261.2022.2147420 36502397

